# Rural inpatient hospitals and substance use—a 10-year retrospective analysis

**DOI:** 10.1007/s11845-025-04095-z

**Published:** 2025-09-30

**Authors:** Sadie Lavelle-Cafferkey, Fintan Sheerin, Catherine Comiskey

**Affiliations:** Miss Sadie Lavelle-Cafferkey, School of Nursing and Midwifery, Trinity College Dublin, Dublin, Ireland

**Keywords:** Alcohol, Hospitals, Rural, Substance use

## Abstract

**Aim:**

To determine the burden and nature of substance use presentations within a defined rural region and provide an estimate of the prevalence and subsequent local needs.

**Method:**

Anonymised secondary data, based on hospital inpatient enquiry (HiPE) records dated 2010–2021 from three sites, were analysed using descriptive and inferential statistics.

**Result:**

Despite similar gender distributions across the three hospitals, approximately 3:1 male to female, substance-related admissions varied significantly across hospitals (*p* < .001). Hospital C had the highest alcohol-related admissions 3537(98.6%), followed by Hospital A for opiates 369(12.3%) and Hospital B for cannabis 161 (2.2%). Only 1151(8.2%) of patients received substance use treatment. Discharge destinations also differed (*p* < .001), with Hospital A having higher patient transfer rates 301(10%) and self-discharge/absconding incidents 415(13.8%) compared to Hospitals B 261(3.6%) and 442(6%) and C 175(4.9%) and 200(5.6%) respectively. Alcohol-related disorders were among the top five non-communicable diseases for men across all sites, and for women in two of the three hospitals, indicating a widespread but gender-variable burden of alcohol-related harm.

**Discussion:**

The data demonstrates significant disparities in substance-related admissions, discharges, and treatment across the hospitals, highlighting the need for integrated care pathways, personalized services, and targeted professional development to address substance use presentations effectively. The findings underscore that a one-size-fits-all approach is insufficient.

## Introduction

Latest international figures show that over 47% of the world’s population live in rural areas [1]. An estimated 2 billion people living in these areas do not have adequate access to essential health services, which adversely affects their health outcomes [2]. Alongside this, it is estimated that 35 million adults have a substance use disorder [3]. Internationally, alcohol is a factor in over 200 diseases and injury related conditions, accounting for over 5.1% of the global burden of disease [4].

In 2015 the United Nations, in conjunction with its member states, developed the 17 Sustainable Development Goals (SDGs), with Health Target 3.5 being to “Strengthen the prevention and treatment of substance abuse, including narcotic drug abuse and harmful use of alcohol” [5]. Following this, in 2017, the United Nations published a policy on ‘The Prevention of Drug use and Treatment of Drug Use in Rural Settings’ [6]. This highlighted that, from a rural perspective, alcohol and substance use is a growing issue internationally, but that, comprehensive data for rural alcohol and substance abuse is not available [6]. A recent systematic review on the impact of Covid-19 on Non-Communicable Diseases (NCD) noted an increase in alcohol consumption and hazardous drinking during the pandemic [7] While this systematic review focused on sub-Saharan African countries, its findings highlighted the importance of undertaking research on this subject in rural areas.

A recent Irish government report indicated that alcohol related admissions in Ireland cost the health service approximately €1.5 billion, or 11.0% of the healthcare budget [8]. The report highlighted an increase in alcohol related discharges with a 3:1 male to female ratio [8]. In relation to substance use, estimated figures from the Irish Department of Health and the Health Research Board (HRB), determined that the treatment cost for substance use in acute hospital settings amounts to €22 million per year [9]. While there is limited research on the prevalence of such use in rural Irish areas, a study on substance use and alcohol in Irish farmers found that nearly 1 in 3 farmers (29%) reported harmful alcohol use, highlighting the need for further research and targeted interventions in rural Ireland [10].

## Methods

The purpose of this study was to determine burden of alcohol and substance use within rural communities by providing an estimate of prevalence and local needs within a defined rural region in Ireland. The Hospital In-Patient Enquiry (HIPE) system is Ireland’s national administrative database for acute public hospital discharges. Each record reflects a single episode of care and is intended to capture hospital activity rather than disease incidence Anonymised secondary data from three rural HiPE datasets (2010 to 2021) were analysed using descriptive and inferential statistics, to explore the trends and associations related to alcohol and substance use in this rural region. The three hospitals analysed in this dataset serve mainly rural populations and are in predominantly rural areas. However, it is crucial to acknowledge that Hospitals A and B serve urban surroundings. The focus of this study was on substance and alcohol use, as defined by the International Classification for Diseases (WHO, 2021) codes F100-F192.

In addition to examining substance use and alcohol, a brief analysis of NCDs, chronic diseases that are not passed from person to person, was undertaken. This additional analysis aimed to further illustrate the burden of care in the three rural hospitals and to provide a more comprehensive understanding of the healthcare challenges within these communities.

A summary of the descriptive and inferential statistics for substance and alcohol use, as well as NCDs, is reported in either table or graph format. Chi-squared tests were undertaken to determine if there were any significant statistical differences between hospitals and genders in terms of the prevalence of substance and alcohol use. A significance level of 0.05 was used for these tests.

## Results

### Number of admissions

**Fig. 1:**
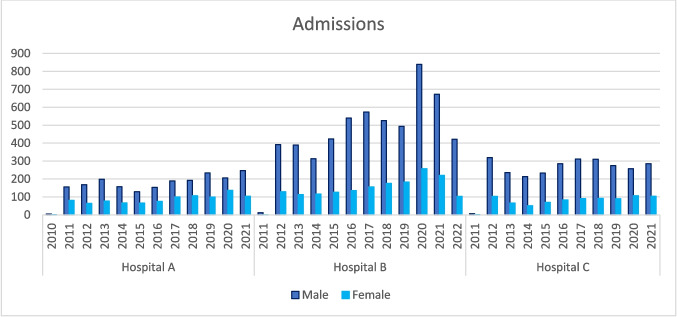
Admissions for 2010–2021 for hospital A, B, and C

Across the three hospitals, 13,906 admission episodes were assigned a diagnosis code relating to alcohol or substance use during the period in question. Figure 1 displays the number of admission episodes broken down by gender and hospital. Although Hospital B had a higher level of activity, the male to female ratios across all three sites were consistent.

To determine if there was a significant difference between genders, a Chi-squared test was conducted. The results showed that 10359 (74%) of admissions were males, whereas 3547(26%) were females, giving a 3:1 male to female ratio. This difference in admission episodes between males and females was found to be statistically significant *p* (< 0.001).

### Most prevalent substance

The main aim of this analysis was to determine which substance was most prominent across the three hospitals. Data pertaining to alcohol, cannabis, sedatives, cocaine, other sedatives, hallucinogens, and multiple substance-related admissions were analysed, with the results presented in Table 1. These results represent admission episodes that were specifically assigned diagnosis codes related to substance use, providing insight into the most common substances associated with hospitalizations in the rural communities.

**Table 1:** Prevalence of substances used

*Substance*	*Hospital A, N %*	*Hospital B, N %*	*Hospital C, N %*	*Totals, N %*	*P*
Alcohol	2698, 89.7%	6962, 95.2%	3537, 98.6%	13,197, 94.9%	< 0.001
Opiate	369, 12.3%	305, 4.2%	23, 0.6%	697, 5%	< 0.001
Cannabis	45, 1.5%	161, 2.2%	33, 0.9%	239,1.7%	< 0.001
Sedatives	17, 0.6%	9, 0.1%	7, 0.2%	33, 0.2%	< 0.001
Cocaine	29, 1%	73, 1%	12, 0.3%	114, 0.8%	< 0.001
Other sedatives	9, 0.3%	13, 0.2%	4, 0.1%	26, 0.2%	0.207
Hallucinogens	3, 0.1%	3, 0.0%	0, 0%	6, 0.0%	0.151

A chi-square test of association found that there was a relationship with admission for alcohol and hospital with hospital C having a greater proportion of alcohol admissions. Overall, 13,197 (94.9%) of admissions with a substance-related diagnostic code across the three hospitals had alcohol recorded. The p-values in the table indicate significant differences between hospitals for most substances, except for "Other Sedatives" and "Hallucinogens. Among the three hospitals, Hospital C had the highest proportion of alcohol-related admissions (98.6%), whereas Hospital B had the highest proportion of cannabis-related admissions (2.2%), and Hospital A had the highest rate of opiate-related admissions (12.3%).

Analyses of other substances, such as opiates, cannabis, sedatives, and cocaine, were undertaken to determine their influence on the hospital facilities and the regions they serve. As seen in Table 1 Hospital A had a significantly higher percentage of opiate-related admissions compared to Hospitals B and C, indicating a higher use of opiates and related health issues in the population served by Hospital A. Other sedatives and hallucinogens did not show any statistical significance.

A chi-square test of association revealed differences in substance distribution by gender across the hospitals, indicating that substance use patterns and treatment-seeking behaviours may vary between males and females in each hospital. While the total number of cannabis-related admissions was 239, Hospital B accounted for 161(67.4%) of these admissions, with males making up 124 of the total cases. This suggests a higher prevalence of cannabis use and related health concerns among males in the population served by Hospital B.

### Impact on the rural hospitals and NCD

While the aim of this research was to estimate the prevalence and local needs regarding alcohol and substance use within the rural area, it is necessary to consider the broader context of alcohol as a significant risk factor for non-communicable diseases (NCDs) in the region. To provide a more comprehensive understanding of the burden on healthcare services, the various conditions patients presented with alongside their alcohol and substance use were analysed. This broader perspective allowed for a more accurate evaluation of the impact on rural healthcare systems and the need for tailored interventions. The findings are presented in Table 2 where a chi square test of association revealed significant differences (p < 0.001) were observed in most disease categories, indicating variations in disease prevalence and healthcare needs across the three hospitals.

**Table 2:** Admissions with NCDs

	Hosp. A, N %	Hosp. B, N %	Hosp. C, N %	*p*
*Male*				
Cardiovascular	448, 22.0%	1965, 35.1%	697, 25.5%	< 0.001
Infectious and parasitic diseases	226, 11.1%	702, 12.5%	136, 5.0%	< 0.001
Neoplasms	52, 2.6%	393, 7.0%	47, 1.7%	< 0.001
Haematology	161, 7.9%	464, 8.3%	127, 4.7%	< 0.001
Endocrine	501, 24.6%	1739, 31.1%	486, 17.8%	< 0.001
Ear	8, 0.4%	41, 0.7%	1, 0.0%	< 0.001
Nervous system	172, 8.5%	822, 14.7%	219, 8.0%	< 0.001
Eye	32,1.6%	122, 2.2%	17, 0.7%	< 0.001
Respiratory	336, 16.5%	1206, 21.6%	415, 15.2%	< 0.001
Digestive	358, 17.6	1468, 26.2%	395, 14.5%	< 0.001
Skin	88, 4.3%	323, 5.8%	63, 2.3%	< 0.001
Musculoskeletal	117, 5.8%	404, 7.2%	97, 3.6%	< 0.001
Genitourinary	132, 6.5%	887, 15.9%	166, 6.1%	< 0.001
Alcohol-related diseases	243, 12.0%	1395, 24.9%	398, 14.6%	< 0.001
*Female*				
Cardiovascular	146, 15.0%	469, 27.4%	152, 17.7%,	< 0.001
Infectious and parasitic diseases	143,14.7%	287, 16.7%	65, 7.6%	< 0.001
Neoplasms	21, 2.2%	117, 6.8%	16, 1.9%	< 0.001
Haematology	95, 9.7%	184, 10.7%	56, 6.5%	< 0.001
Endocrine	268, 27.5%	462, 27.0%	163, 19.0%	< 0.001
Ear	5, 0.5%	18, 1.1%	2, 0.2%	< 0.046
Nervous system	61, 6.3%	243, 14.2%	45, 5.3%	< 0.001
Eye	6, 0.6%	35, 2.0%	1, 0.1%	< 0.001
Respiratory	117, 12.0%	346, 20.2%	127, 14.8%	< 0.001
Digestive	156, 16.0%	422, 24.6%	119, 13.9%	< 0.001
Skin	36, 3.7%	93, 5.4%	11, 1.3%	< 0.001
Musculoskeletal	78, 8.0%	147, 8.6%	35, 4.1%	< 0.001
Genitourinary	120, 12.3%	268, 15.6%	79, 9.2%	< 0.001
Alcohol-related diseases	118, 12.1%	370, 21.6%	158, 18.4%	< 0.001

The gender analysis of this data found that except for female patients in Hospital A, alcohol-related disorders were among the top five NCDs across all hospitals and patient groups in this dataset. This highlighted the significant impact of alcohol consumption on overall health and the burden it places on healthcare systems.

Hepatitis, liver cirrhosis, and hepatic failure were the most prevalent alcohol-related disorders among patients of all genders in each hospital. Interestingly, male patients exhibited a higher prevalence of NCDs compared to females, particularly in Hospital B.

### Discharge and treatment

Understanding discharge destinations is crucial in identifying areas where additional resources might be needed, such as other healthcare facilities or community support. The data revealed that across all three hospitals, most patients were discharged to their homes. However, a Chi-square test of association revealed a significant number of patients in Hospital A had different discharge outcomes: 415(13.8%) self-discharged or absconded (*p* < 0.001), and 301(10%) were transferred to another hospital. These findings demonstrate the varying discharge needs and outcomes for patients admitted with alcohol or substance use-related issues across the three hospitals Table 3.

**Table 3:** Discharge destination

Gender	Discharge Code	Hosp. A, N %	Hosp. B, N %	Hosp. C, N %	*p*
Male	Home	1339, 65.9%	4469, 79.9%	2088, 76.5%	< 0.001
	Healthcare Setting in the Community	117, 5.8%	351, 6.3%	265, 9.7%	< 0.001
	Transfer to Another Hospital	219, 10.8%	189, 3.4%	135, 4.9%	< 0.001
	Prison	6, 0.3%	4, 0.1%	0, 0.0%	< 0.001
	Self-discharge or Absconded	303, 14.9%	355, 6.3%	168, 6.2%	< 0.001
	Died in Hospital	38, 1.9%	188, 3.4%	68, 2.5%	< 0.001
	Temporary Place of Residence	11, 0.5%	40, 0.7%	6, 0.2%	< 0.001
Female	Home	704, 72.1%	1372, 80.0%	671, 78.3%	< 0.001
	Healthcare Setting in the Community	55, 5.6%	99, 5.8%	85, 9.9%	< 0.001
	Transfer to Another Hospital	82, 8.4%	72, 4.2%	40, 4.7%	< 0.001
	Prison	0, 0.0%	1, 0.1%	0, 0.0%	< 0.001
	Self-discharge or Absconded	112, 11.5%	87, 5.1%	32, 3.7%	< 0.001
	Died in Hospital	17, 1.7%	68, 4.0%	29, 3.4%	< 0.001
	Temporary Place of Residence	6, 0.6%	15, 0.9%	0, 0.0%	< 0.001

Significance levels: *p < 0.05: **p < 0.01: ***p < 0.001

*ns* not significant

The main treatment provided to patients was alcohol detoxification, however this was limited with the highest prevalence of 580(28.3%) of males receiving alcohol detoxification in Hospital A. While 13,906 presentations for alcohol or substance use were reported, it is striking that only 1151(8.2%) of these received any form of treatment, resulting in 12,755 (91.8%) of individuals in this dataset not receiving any formal treatment for their alcohol or substance use. Formal treatment, as captured by procedure codes, included rehabilitation, detoxification, counselling, and substance use assessments.

## Discussion

This analysis of HiPE data sheds light on hospital activity with a focus on alcohol and substance use. Key findings pertain to admissions, non-communicable diseases (NCDs), alcohol-related disorders, discharges, and access to treatment.

While alcohol emerged as the primary substance identified, further analysis revealed the significance use of other substances. Variations in opiate-related admissions among hospitals might be due to their locations, as Hospitals A and B serve urban populations, despite being in an overall rural setting in Ireland, while Hospital C serves predominantly rural populations. These results align with a 2018 study indicating lower opiate prevalence rates in the county where Hospital C is located [11]. Hospital A recorded the highest admissions related to opiates for both males and females. Similarly, Hospital B, serving an urban area with a large student population, had the highest cannabis-related admissions, consistent with Irish data highlighting cannabis use among 15–34-year-olds [12]. Despite the rising attention on cannabis use across Europe [13], alcohol remains the dominant substance associated with rural in-patient hospital admissions in Ireland, underscoring the need for a comprehensive approach addressing opiates, cannabis, and alcohol.

With the exception of women in hospital A, alcohol-related disorders ranked among the top five NCDs in this dataset, demonstrating the substantial impact of alcohol on individual health. The World Health Organization (WHO) recognizes alcohol as a significant risk factor for NCDs and has incorporated it into the Global Action Plan for NCD prevention and control [14]. These findings, which align with a narrative review linking alcohol to cardiovascular disease, endocrine disorders, and liver disease [15], emphasize the importance of addressing alcohol use in NCD prevention and treatment strategies, through integrated care when a patient is admitted to an acute general hospital.

Understanding discharge destinations is crucial for identifying potential resource allocation needs, such as additional healthcare settings or community support. This analysis emphasizes the distinct challenges encountered by each hospital, alongside the lack of follow up care, underscoring the importance of establishing integrated care pathways that facilitate seamless patient transitions across various healthcare settings and community resources.

The majority of patients from all three hospitals were discharged to their homes, aligning with national averages [16]. However, a significant number of patients either were transferred from Hospital A to another hospital, self-discharged or absconded. This highlights the necessity for integrated care pathways within the community and across healthcare settings.

Despite the evident need for alcohol use treatment, access was limited, with alcohol detoxification being the primary treatment provided, and principally to males in Hospital A. This finding is particularly concerning given the substantial evidence demonstrating that rural populations face disparities in accessing treatment compared to their urban counterparts [17]. Rural populations often encounter additional barriers, including limited services, increased travel times, and stigma when attempting to access care [17].

## Conclusion

This analysis of rural inpatient hospitals highlights alcohol as the primary substance of concern, linked to non-communicable diseases and impacting acute hospital care. Opiate and cannabis use also warrant attention in these settings. Each hospital's unique profile calls for adaptable, integrated care solutions tailored to rural areas, and extension of the current pilot sites for integrated alcohol services operated by the HSE [18]. Addressing disparities in treatment access and improving services is crucial for supporting the well-being of these communities. A comprehensive and tailored approach is essential to effectively address substance-related admissions in rural hospitals.

### Limitations

This study relies on HIPE data, which capture only administrative and diagnostic information from acute public hospital discharges. The dataset does not include details such as clinical notes, community-based presentations, or repeat attendances by the same individual across facilities. As such, the findings may underestimate the broader burden of substance-related harm and limit insight into patient trajectories over time.
